# Assessing sugar intake rapidly – a short form of the Marburg Sugar Index (MSI)

**DOI:** 10.1186/s12903-023-03403-2

**Published:** 2023-09-29

**Authors:** Jutta Margraf-Stiksrud, Klaus Pieper, Renate Deinzer

**Affiliations:** 1 Department of Psychology, Philipps University of Marburg, Gutenbergstrasse 18, D-35032 Marburg, Germany; 2 Centre for Dentistry, Oral and Maxillofacial Medicine, Philipps University of Marburg, Georg-Voigt-Strasse 3, D-35033 Marburg, Germany; 3 Department of Medical Psychology, Department of Medicine, Justus-Liebig-University Giessen, Klinikstr. 29, D-35392 Giessen, Germany

**Keywords:** Oral health, Dietary sugars, Nutrition survey, Nutritional habits, Food frequency, Population based survey

## Abstract

**Background:**

Sugar intake is a major nutritional factor in the development of dental caries. To further clarify its contribution to oral health-related diseases, population-based investigations are recommended. To facilitate economic and reliable assessment of sugar intake, a short form of the approved Marburg Sugar Index (MSI) was developed.

**Methods:**

According to the principles of item reduction based on original data, a six-item-short form was constructed. A total of 468 participants (aged 15–81) answered the short form together with the long form in a counterbalanced cross-over design, and with two questionnaires concerning self-efficacy and decisional balance in oral health to verify construct validity.

**Results:**

Comparable item characteristics to the original MSI and a high correlation with the long form prove the usefulness of the short form, which was processed by the participants in less than one minute. Low correlations to the other two constructs show discriminant validity.

**Conclusion:**

The new short form of the MSI (MSI-S) can replace the long form, especially in population-based studies with no restrictions on assessment quality but with sufficient time saved to add other variables necessary to explore oral health-related issues.

## Introduction

The frequency of sugar intake is a relevant factor in the development of caries in children, adolescents, and adults [1–4]. Since the WHO recommendations for restriction of sugar intake were published in 2015 [5] (from < 10% to < 5% for additional health effects), several studies confirmed the benefits of reduced frequency of sugar intake for oral health [6, 7]. To estimate the frequency of sugar intake in population-based studies, questionnaires and food frequency lists are useful as methods which are economic and easy to administer. Recently, the Marburg Sugar Index (MSI) was proposed as a reliable and valid instrument to assess these nutritional habits in children (aged 6 to 12) and their parents in large groups [8, 9]. Participants should estimate the consumption frequency of food and drinks relevant to oral health in six different situations. The study results showed good internal consistency of the instrument and correlations with relevant aspects of oral health. However, in the study of Schmidt et al. [9], a data analysis had been separately performed for n = 429 participants (parents and their 12-year-old children) with full records and n = 400 participants with missing data (up to two items for 85%). Hence, a considerable proportion of missing data in both age groups indicates that subjects tend to reduce the workload by skipping some items, perhaps finding it too tedious to finish answering a lot of similar questions.

Therefore, it appears desirable to simplify the evaluation procedure by reducing the amount of food items, which will lead to a shorter questionnaire with comparable psychometric potential and to a similar useful sugar index. Then, this even more practical and reliable instrument could be applied population-wide, e.g., epidemiological, studies without producing notable missing data. Hence, the current investigation aimed to develop a short form of the approved MSI and present data to proof construct validity. There are several ways to develop short forms of established instruments. For the choice of the reduction method, it is decisive which aspects and properties of the original instrument are to be retained. In the case of multidimensional questionnaires, the retention of the dimensions is often important; sometimes certain item contents are particularly relevant in order to represent a construct appropriately in a short form. For the widely used OHIP, for example, various short forms have been developed [10–12]. Their item composition differs depending on the focus selected for item reduction. The current MSI focuses on situational contexts when asking about food consumed. It is therefore a situation-response-questionnaire. The short form should retain precisely this characteristic. The reduction of items was performed along this aim and a validation study was run to test the hypothesis that the short form would lead to results comparable with the long form.

## Materials and methods

### Development of the short form

The original MSI is based on 53 items that were constructed according to relevant principles for measuring nutritional habits [13, 14]. Participants have to estimate the frequency of intake of special food items in six situations (eating between meals, eating bread, drinking while not at home, eating breakfast, eating while watching TV, eating while not at home). Participants can indicate the frequency of their consumption by selecting one of five options: never, seldom, occasionally, often, always. For each situation, several items that are positive, neutral, and negative for oral health are presented. They add as follows: Eating between meals – 7 items, eating bread − 7 items, drinking while not at home − 10 items, eating breakfast − 8 items, eating while watching TV − 10 items, eating while not at home − 11 items. Additionally, participants can add food items to the list for each situation. Within the 53 items, 25 named food and drinks that are detrimental to oral health due to their composition and/or texture. Table 1 shows all these 25 items listed under the respective situation (in italics). The MSI is the sum value of these 25 items, when „never“ is assigned a score of 0, and „always“ is assigned a score of 4, with the others in between.

### Reduction of items

Based on the original study [15] with data of more than 3000 adults (parents of the surveyed children), only those food items were inspected that are detrimental for oral health, as in the original MSI. The following principles guided the further process of item reduction: The variety of situational contexts should be maintained, as they are relevant for discovering detrimental sugar intake. Furthermore, the new short form should be able to differentiate between various degrees of unfavorable nutritional habits, in other words, the items should produce as high a range of answers as possible. Finally, the resulting items should largely represent the content of the original scale. Hence, for each of the six nutritional situations, those items with the largest variance in the study population and with low frequencies of missing data (below 10%) were chosen. Next, the item with the highest item-total correlation in those participants with no missing data was identified, resulting in six items (one per situation) with item-total correlations above 0.30. As a preliminary last step, the sugar index estimated with these six items was correlated with the original index based on the complete questionnaire of the participants, resulting in r = 0.83.

## Empirical study: Assessment of the psychometric properties of the shortened questionnaire

### Ethics

This study was conducted in accordance with the Declaration of Helsinki and ethical permissions were granted by the Ethics Board of the Medical Faculty at the University of Giessen, Germany (No. 233/21). Informed consent was obtained from all study participants or their legal guardians (if under 16 years of age). The participants were invited via a mailing list of the University of Giessen and a private list, which was only addressed to adults. In the mail they were informed about the aims and the procedure of the study and parents were asked to forward the mail to their children if they agreed with their participation. Recipients of the mail who wished to participate were able to open the online survey via a link in the mail. On the survey home page, they were again informed about the objectives of the study and privacy. Participation was anonymous and participants could end their participation at any time (by closing the browser window).

### Procedure

To assess the convergent validity of the short form with the long form and its further psychometric properties (internal consistency and item characteristics), an online survey was conducted where the short and the long form were presented in a counterbalanced cross-over design. Between the two forms, two distracting questionnaires (decisional balance [16, 17] and self-efficacy expectations regarding oral hygiene behavior [18] were presented, to minimize recall of the answers given first when answering the second form. These questionnaires were also used to assess the discriminant validity of the sugar index questionnaire (see below). Thus, one version of the survey presented the short form first, then the two other oral health-related questionnaires, and then the long form. The other version began with the long form and presented the short form after participants had answered the distracting questionnaires. The two versions of the survey were presented in random order (half of the participants answered the long version first, half the short version). To avoid missing data, participants only could go through the questionnaires when every item was processed. Finally, participants answered questions to gather demographical data (gender, age, education). Email invitations to participate in this survey were sent using university mailing lists in Giessen and private lists, requesting further distribution.

### Assessment of discriminant validity

The distracting questionnaires represent the following constructs: self-efficacy expectations regarding tooth brushing (SE brush), self-efficacy expectations regarding approximal hygiene (SE approx.), decisional balance (pros vs. cons) of brushing and approximal hygiene (pro brush, cons brush, pros approx., cons approx.). These constructs are expected to be fairly independent of nutritional behavior though also related to oral health. Thus, they are well suited to assessing discriminant validity.

### Statistics and study size

The minimum study size was set a priori to 100 participants, as it is known that the descriptive data assessed here obtain a good precision (small confidence intervals) with this study size. To describe psychometric aspects of the newly developed short form of the MSI, means, variances, item-total correlations and Cronbach’s alpha were computed. To assess its construct validity, the correlations with the original form and the two other oral health questionnaires were determined. The normal distribution of the questionnaire data was assessed by visual inspection as suggested for large sample sizes. To control for the possible confounding effects by the sequence of the presentation of the two questionnaire forms (long–short vs. short–long), by gender and by dropout, t-tests for independent measures were run. Cohen’s d was computed as a measure of effect size.

## Results

During winter 2021/2022, 573 individuals started the survey, while 468 completed all questionnaires (82% females, 15–81 years old, mean age 30.4 years). When participants started with the long form, 17.5% dropped out while answering the questionnaire. Dropout rate for starters with the short form was 7.0%. Based only on fully completed questionnaires, mean times for answering the long form were 649.6 s (long form at the beginning of the questionnaire) and 222.3 s (long form at the end of the questionnaire). For the short form, the corresponding values were 39.0 s and 31.1 s, respectively.

### Descriptive statistics of the two forms

Means for the 25 items of the long form were 1.22 (minimum) and 2.63 (maximum), and for the 6 items of the short form, they were 1.62 (minimum) and 2.54 (maximum). The standard deviations as an indicator for varying estimations of food consumption frequencies in the study group resulted in SD = 0.53–1.17 (long form) and SD = 0.80–1.04 (short form). Item-total correlations (part–whole corrected) produced scores between r = 0.119 and r = 0.584 (long form), and scores from r = 0.203 to r = 0.414 (short form), respectively (for details, see Table 1).

**Table 1:** Item characteristics of MSI (long and short form) (n = 468)

Foods	Long form^1^			Short form		
Item	Mean	Standard Deviation	Item-total correlation	Mean	Standard Deviation	Item-total correlation
*1. Eating between meals…*						
Bananas	2.60	1.031	0.179			
Chocolate bars	2.63	1.035	0.545			
Cake	2.32	0.831	0.489			
Granola bars	1.78	0.850	0.358			
Fruit yoghurt	1.80	0.932	0.367	1.62	0.797	0.276
*2. Eating bread…*						
Sweet rolls	1.93	0.869	0.410	2.19	0.974	0.337
*3. Drinking while not at home…*						
Fruit juice	1.75	0.850	0.345	1.71	0.857	0.203
Iced tea	1.47	0.738	0.347			
Coffee with sugar	1.36	0.788	0.155			
Soda/Cola	2.00	0.979	0.400			
*4. Eating breakfast…*						
Cornflakes with milk	1.70	0.995	0.342	1.72	0.980	0.369
Muesli with fruit	2.30	1.171	0.119			
Rolls and jam	2.27	1.019	0.299			
Cake	1.54	0.714	0.433			
Fruit yoghurt	1.57	0.859	0.387			
*5. Eating while watching TV…*						
Sweet popcorn	1.41	0.640	0.383			
Chips	2.33	0.936	0.296			
Sour apple rings	1.28	0.600	0.227			
Chocolate	2.59	1.065	0.507	2.54	1.037	0.325
Dried apricots/dates	1.41	0.754	0.154			
Cookies	2.14	0.959	0.584			
*6. Eating while not at home…*						
Chocolate bars	2.12	1.003	0.563			
Cake	1.71	0.811	0.524			
Fruit yoghurt	1.22	0.528	0.273			
Cookies	1.95	0.936	0.515	2.08	0.934	0.414

^1^Instruction for all situations: “How often do you eat (drink) the following when…”

### Reliability statistics

Cronbach’s alpha resulted in alpha = 0.828 (long form) and alpha = 0.584 (short form). When we used Spearman–Brown correction for the reduced amount of items, the new short form produced an alpha of 0.846. The visual inspection of frequency graphs revealed normal distribution for the two MSI scores. The Pearson correlation between both forms was r = 0.789 (n = 468).

### Discriminant validity statistics

The other questionnaires (decisional balance and self-efficacy) were strongly skewed; therefore, Spearman correlations between these and the two MSI forms were computed. These were valued between r = 0.126 and r = -0.121 for the long form, and between r = -0.108 and r = 0.144 for the short form (Table 2).

**Table 2:** Correlation of Sugar Indices with self-efficacy and decisional balance^1^

	SE brush	SE approx.	Pros brush	Cons brush	Pros approx.	Cons approx.
MSI long	-0.121	-0.108	0.126	0.099	0.083	0.114
MSI short	-0.108	-0.093	0.033	0.063	0.022	0.144

^1^ SE brush—self-efficacy brushing; SE approx. —self-efficacy approximal hygiene; Pros brush—pros of brushing; Cons brush—cons of brushing; Pros approx.—pros of approximal hygiene; Cons approx.—cons of approximal hygiene

### Sensitivity analyses

T-tests revealed no significant differences between the two presentation sequences of the questionnaire forms (all p > 0.09, all d < 0.31). Data of participants who dropped out did not differ significantly from those who did not (both, regarding the long and the short form, all p > 0.29, all d < 0.27). No difference between the scores of male and female participants was found for both forms (all p > 0.07, all d < 0.19). The correlations with age were significant but very low (r = -0.163 long form, r = -0.179 short form).

## Discussion

The data concerning the newly developed short form of the Marburg Sugar Index (MSI) were based on the responses of more than 500 adults. Thus, the results are appropriately reliable. As expected, the short form (MSI-S) with six items produces psychometric indicators comparable to those of the original long form. The means were similar, standard deviations showed only a small, but self-evident, reduction in variance, and all but one item-total correlation was around r = 0.30 or higher. When corrected for the reduction of items, the Cronbach’s alpha of the MSI-S is as good as that of the original MSI. Standing alone, MSI-S can achieve valuable results with a consistency of r = 0.58, which is fully acceptable, especially in large population surveys, e.g., epidemiological studies, which is the intended field of application. As a sign of content validity, the moderate score of consistency is comprehensible as it represents actual answers from six different nutrition situations. All items concern foods that are consumed in everyday life by nearly everybody, but just with different frequency. This can be valued as a strength of the questionnaire.

The short form not only has similar psychometric properties as the original MSI; the convergent validity with the original is also good, as can be seen by the high correlation of the two scales. To estimate the discriminant validity of the two forms with regard to other oral health-related constructs, the correlations with oral hygiene-related self-efficacy expectations and decisional balance were assessed (Table 2). The negligible correlations confirm a good discriminant validity in this respect.

Depending on the online presentation sequence, 17.5% and 7% of the study group, respectively, did not answer the long form and short form completely. However, no one dropped out while answering the short form. Thus, a main objective was met, i.e., the reduction of dropouts by reducing the number of items. Furthermore, participants answered the MSI-S on average in under one minute of processing time. This renders the questionnaire particularly suitable for use together with other instruments or within a questionnaire battery, as it is often necessary in population-based surveys.

When used to assess the correlation between dental outcomes, especially caries experience, a methodological economic and valid exploration of dietary habits is necessary. It should focus on those foods which are relevant for changes in the chemical homeostasis of the oral cavity. Covering general intake of food to gain also information about other possible health risks (e.g. obesity) is less likely to contribute to a clarification of the complex correlation between dental diseases and sugar intake [2, 13, 14]. Often used so called food frequency lists [19] mostly are not specific enough. Therefore, focusing on food items with relevance for dental health along with common consumption in specific situations of intake, i.e., between meals is as mandatory as asking about frequency of intake. For caries development, frequency seems to be more relevant than the total amount of sugar intake [20, 21]. The correlation of sugar intake and caries experience is part of a complex mutual dependency of oral hygiene, fluoridation, socio-economic status, and oral health [21]. Consequently, a multiple set of variables has to be taken into consideration and is to be measured in future studies of oral health and sugar intake. The new short form of the MSI with only six items can be highly useful for these purposes.

In those studies, the validity of the MSI-S should be further monitored. Objective assessment of dietary habits in everday life as the most important criterion is discussed (e.g. biological markers, diaries of food intake [3, 22]. Appropriate methods should be based on both the object of investigation and the respective research question. Therefore, to proof the criterion validity of the MSI-S in dental contexts, oral health would be an important criterion of the usefulness of MSI. For the long form, this was already confirmed in children with the dmft-index [8, 9]. For the short form, data in this regard are gathered in a currently performed epidemiological study and will be reported.

The present study has its strengths in the data-driven methodology of item reduction of the original instrument. The study population was large enough to allow for firm conclusions regarding the internal consistency and convergent and discriminant validity of the instrument. However, the composition of the study population represents a major limitation. The majority were young female adults. By increasing the variance within the sample, one would also expect correlations to increase. Thus, the correlational analyses in the present sample rather underestimate than overestimate the correlations in the general population. Such population studies are also needed to explore whether the MSI-S contributes to variance explanation of caries distribution. The original MSI, with its considerable dropout rates and long answering time, was problematic with respect to its use in panel studies. The short form is more suitable for such studies. Future research should apply this instrument to further explore the relationship between frequency of sugar consumption and caries development.

## Data Availability

The data presented in this study are available on reasonable re-quest from the corresponding author. The data are not publicly available due to general data protection regulations.
